# Ethical challenges in the clinical translation of anti-amyloid monoclonal antibodies for Alzheimer’s disease: a scoping review

**DOI:** 10.3389/fpsyt.2026.1953954

**Published:** 2026-10-05

**Authors:** Alexandra Wicker, Amanda Courtright-Lim, Jon Tilburt

**Affiliations:** 1 Biomedical Ethics Research Program, Mayo Clinic, Phoenix, AZ, United States; 2 Department of Internal Medicine, Mayo Clinic, Phoenix, AZ, United States

**Keywords:** Alzheimer’s disease, anti-amyloid monoclonal antibodies, anti-amyloid therapy, bioethics, ethics

## Abstract

**Background:**

The development and evaluation of anti-amyloid beta monoclonal antibodies (mAbs) for the treatment of Alzheimer’s disease have been accompanied by extensive ethical debate spanning clinical trials, regulatory processes, prescribing practices, and healthcare delivery. This scoping review examined literature published between 2006 and 2026 to identify and clarify the landscape of ethical considerations surrounding the implementation, prescribing, and delivery of anti-amyloid mAbs across both research and clinical settings.

**Methods:**

We searched PubMed, Scopus, Web of Science, Embase, and Ovid EBM Reviews (including CENTRAL and CDSR) from 2006 to March 2026 using a structured search strategy. Eligible publications focused on ethical issues associated with the clinical translation of anti-amyloid mAbs. Two reviewers independently screened titles, abstracts, and full-text articles, with duplicate extraction performed on an initial subset of publications. The review was conducted using JBI guidance and reported according to PRISMA-ScR.

**Results:**

We identified 84 publications from 23 countries published between 2014 and 2026, including 12 original research studies, 1 systematic review, and 71 commentaries, narrative reviews, legal analyses, or practice guidelines. Analysis identified 32 subthemes, which were synthesized into 7 overarching themes: (1) beneficence and nonmaleficence, (2) respect for persons, (3) equity and accessibility, (4) evidence and trial integrity, (5) governance and stewardship, (6) relational and social impacts, and (7) health system readiness and sustainability. Among the 32 subthemes, uncertain clinical benefit and risk of harm (both within the beneficence and nonmaleficence theme) were discussed most frequently within the literature, each appearing in 61 publications.

**Conclusions:**

The biomedical literature documents a broad range of ethical considerations relating to anti-amyloid mAbs. Despite this growing literature, it is primarily based on commentary, with few empirical studies, and even fewer incorporating the perspectives of patients, families, and clinicians. Doing so may add further context and nuance to ethical discussion about mAbs in the years ahead.

## Introduction

Alzheimer’s disease (AD) is a progressive neurodegenerative disorder that significantly impairs cognitive and functional abilities. Although its etiology is not entirely understood, AD is in part related to the accumulation of amyloid beta (Aβ) plaques and tau neurofibrillary tangles within the brain (1). Among the many proposed theories of AD pathogenesis, the amyloid cascade hypothesis has been particularly influential, positioning Aβ as a central therapeutic target (2). Consequently, several iterations of anti-amyloid monoclonal antibodies (mAbs) have been developed over the last two decades in pursuit of an effective disease-modifying therapy for AD (3). The U.S. Food and Drug Administration (FDA) has approved three anti-amyloid mAbs to date, including aducanumab, lecanemab, and donanemab, marking a major shift in the therapeutic landscape for AD. Unlike symptomatic treatments, these therapies seek to modify the underlying disease process, offering new possibilities for slowing cognitive decline.

Nevertheless, the search for a disease-modifying therapy for AD has long been accompanied by ethical debate. Previous reviews have synthesized ethical issues related to AD and dementia research more broadly, particularly regarding the enrollment of cognitively impaired individuals in clinical trials and concerns surrounding informed consent, vulnerability, decision-making capacity, exposure to harm, and social value (4–6). However, the development of anti-amyloid therapies has introduced additional ethical considerations beyond research participation. These therapies require monitoring for amyloid-related imaging abnormalities (ARIA), necessitating serial magnetic resonance imaging (7–9). Moreover, because carrying one or two copies of the APOE ϵ4 allele is associated with an increased risk of ARIA, genetic testing is recommended before beginning treatment, raising downstream implications for counseling and family members (8–11). Although subcutaneous formulations are emerging, including a subcutaneous formulation of lecanemab approved by the FDA in July 2026, drug delivery has historically relied on specialized infusion infrastructure. Taken together, these features require careful weighing of what many deem modest clinical benefits against potential inequities, burdens, and added risks.

Ethical scrutiny intensified following the FDA’s approval of aducanumab in 2021 despite contradictory trial results and opposition from members of the Peripheral and Central Nervous System Drugs Advisory Committee, several of whom subsequently resigned (12, 13). The approval generated substantial discourse regarding clinical benefit, patient safety, and healthcare resource allocation, with discussions of evidentiary standards and regulatory reform circulating more broadly. Although aducanumab’s FDA approval was formally withdrawn in November 2024 following Biogen’s decision to discontinue its commercial development, the ethical questions raised during its approval have persisted. Subsequent approvals of lecanemab and donanemab have shifted attention toward the responsible implementation of anti-amyloid therapies in routine clinical practice, raising questions surrounding clinician obligations and health system readiness.

To our knowledge, no systematic summary of the ethical issues associated with anti-amyloid mAbs exists in the biomedical literature. Consequently, existing scholarship remains dispersed across empirical studies, policy papers, normative analyses, and clinical guidelines, making it difficult to map the range of published ethical considerations surrounding these therapies. We sought to identify the major ethical issues discussed in the biomedical literature and highlight areas requiring further ethical analysis as anti-amyloid mAbs continue to be developed and implemented in clinical practice.

## Methods

This scoping review was conducted and reported in accordance with the Preferred Reporting Items Systematic reviews and Meta-Analyses extension for Scoping Reviews (PRISMA-ScR) (14). An *a priori* review protocol was developed before study commencement in consultation with a research librarian and was used to guide the search strategy, eligibility criteria, and data extraction procedures. The protocol was not formally registered as registration is not a required component of scoping review methodology, and it was maintained as an internal working document throughout the study.

### Research questions and PCC framework

The overarching aim of this review was to synthesize the available literature on the ethics of anti-amyloid mAbs for Alzheimer’s disease. To ensure methodological clarity, the review was conducted according to the Joanna Briggs Institute (JBI) Manual for Evidence Synthesis and structured using the PCC (Population, Concept, Context) framework (15, 16). The population (P) comprised individuals with Alzheimer’s disease in any stage, including those with preclinical disease. The concept (C) was defined as ethical considerations surrounding the translation, prescription, and administration of anti-amyloid mAbs within the context (C) of clinical practice and biomedical research. Ethical considerations were broadly defined as discussions involving ethical principles, values, norms, obligations, trade-offs, or implications.

### Information sources and search strategy

We conducted a comprehensive literature search across five electronic databases: PubMed, Scopus, Web of Science, Embase, and the Ovid EBM Reviews platform (including the Cochrane Central Register of Controlled Trials [CENTRAL] and the Cochrane Database of Systematic Reviews [CDSR]), covering publications from 2006 through 3 March 2026. This timeframe was selected because the first anti-amyloid mAb, bapineuzumab, was developed in the early 2000s and entered Phase III trials in 2007 (17). The search strategy was developed in consultation with an institutional research librarian and employed both Medical Subject Headings (MeSH) and free-text terms. The general search string applied across all databases was:

(“ethic” OR “ethics” OR “moral*” OR “justice” OR “benefit*” OR “harm*”) AND (“Alzheimer” OR “Alzheimer’s” OR “dementia”) AND (“anti-amyloid” OR “amyloid-related” OR “recently approved” OR “lecanemab” OR “donanemab” OR “aducanumab” OR “monoclonal antibod*” OR “passive immunotherapy” OR “biologic therapy”).

Full search strategies as well as the number of records identified for each database are provided in **Supplementary Appendix A**. The formal search was supplemented with articles manually identified through the reference lists of included publications.

### Eligibility criteria and publication selection

Title and abstract screening, full-text review, and data extraction were conducted in May 2026. Eligible publications included empirical and non-empirical peer-reviewed research focusing on ethical issues associated with the clinical translation of anti-amyloid mAbs. Sources were included if they: (i) discussed ethical considerations related to the translation, prescription, or administration of anti-amyloid mAbs; (ii) focused on individuals with Alzheimer’s disease in any stage; (iii) were published in English between 2006 and 3 March 2026; and (iv) had an available full text. Publications were restricted to English due to the language capabilities of the review team and the lack of resources for translation and screening of non-English publications. Publications focused exclusively on regulatory or reimbursement processes were excluded when they did not address ethical implications for healthcare delivery, clinical decision-making, or research. Publications examining regulatory issues such as accelerated approval pathways, surrogate endpoints, or evidentiary standards were included when these issues were considered in relation to healthcare or research ethics. No restrictions were placed on geographical setting or study design.

All retrieved records were first imported into EndNote and then Covidence, which were used to support duplicate detection and screening management, respectively. Title, abstract, and full-text screening were conducted independently by two reviewers (AW and ACL), with disagreements resolved through discussion until consensus was reached.

### Data extraction and synthesis

Data extraction was conducted using a template created in Covidence. Data extraction for an initial subsample of 16 publications was performed independently by two reviewers (AW and ACL) working in parallel. Extracted variables included publication year, country of first author, study design, context, depth of ethical reasoning, ethical frameworks, principal findings, and ethical themes. Depth of ethical reasoning was categorized according to the extent to which ethical arguments were developed and classified as either implicit discussion of ethical issues or explicit engagement with ethical theories, principles, frameworks, or normative analysis; this is denoted in **Supplementary Appendix B**. Ethical frameworks were identified based on either explicit reference by source authors or reviewer-assessed implicit ethical reasoning reflected in the publication. When an article drew upon more than one ethical framework, all applicable frameworks were recorded; therefore, framework categories were not mutually exclusive, and individual articles could be assigned to multiple categories. Framework classifications were assigned using the operational definitions provided in **Supplementary Appendix C**. Ethical subthemes and themes were compiled inductively and refined iteratively throughout the extraction process. Themes were coded at the publication level, such that a publication was considered to discuss a theme when it included at least one substantive discussion of that theme. Themes were not treated as mutually exclusive, and publications could be assigned to multiple themes when applicable. Extracted data were compared across reviewers for the first 16 articles, and discrepancies were resolved through discussion. Once clarity and consistency of extraction fields had been achieved, one reviewer (AW) extracted and coded the remaining publications, with consultation from a second reviewer (ACL) when uncertainties arose.

Consistent with JBI guidance for scoping reviews, formal methodological quality appraisal of included sources was not undertaken because the objective was to map and characterize the existing literature rather than assess study quality.

### Ethical considerations

Ethical approval was not required for this study as no primary data were collected and no human participants were involved.

## Results

The screening and study selection process is presented in **Figure 1**. In total, 84 publications fulfilled the eligibility criteria and were included in the review (7, 8, 18–99). A full list of the selected publications and their characteristics is available in the **Supplementary Appendix**.

**Figure 1:**
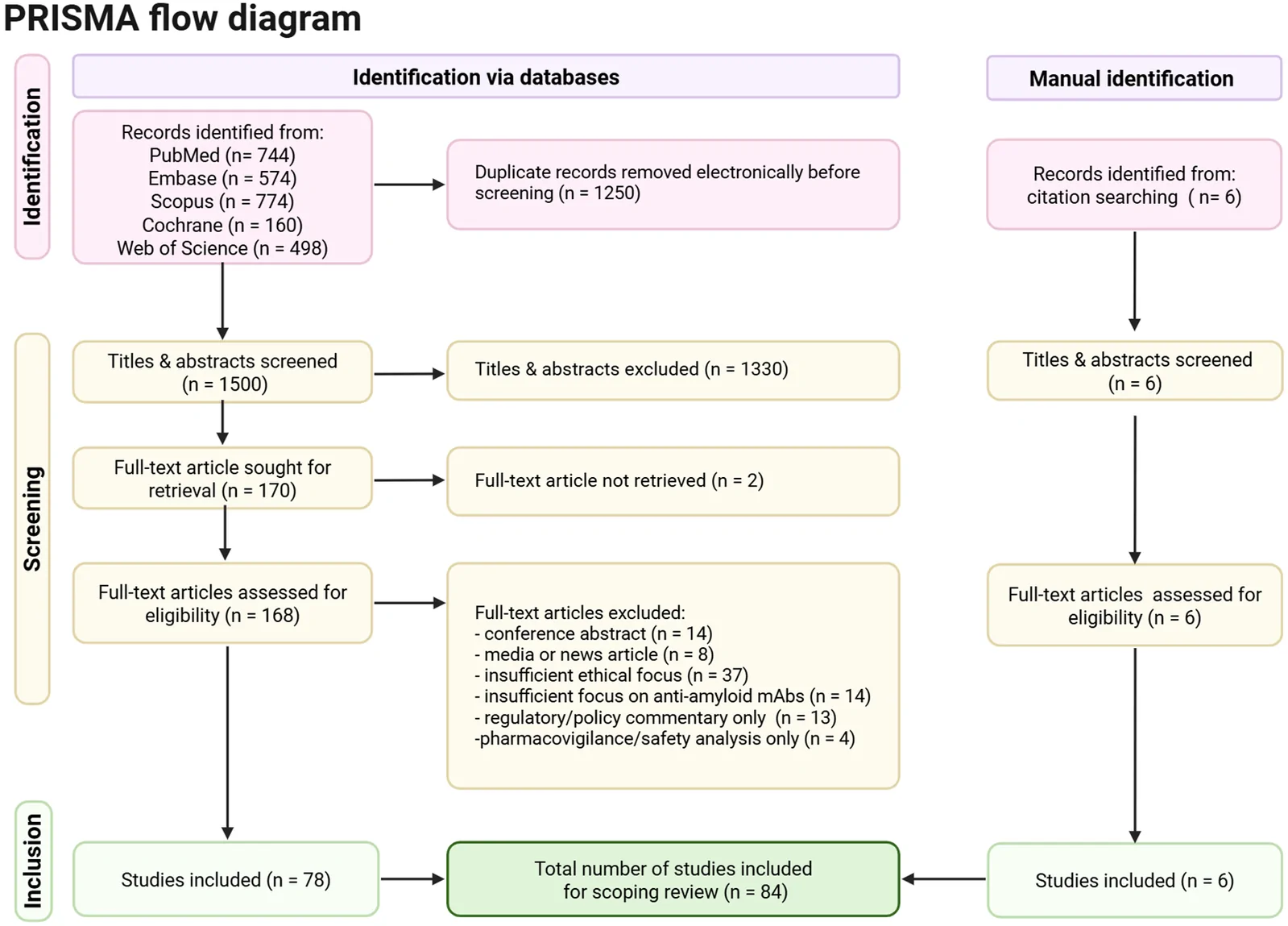
PRISMA-ScR flow diagram. Flow diagram depicting the search, screening, and selection process used to identify sources of evidence included in this scoping review.

### Characteristics of included publications

Publications spanned 2014-2026, showing a marked increase in recent years. The majority (*n* = 62) were published from 2023 onward, with the highest annual frequency recorded in 2025 (*n* = 22), followed by 2024 (*n* = 18) and 2023 (*n* = 16). Publications were sparse prior to 2020, with only 2 publications in 2016 and single publications in 2014 and 2017.

The first authors of included publications were affiliated with institutions in 23 countries, with the United States accounting for 46.4% of the sample, followed by France/Argentina (6%), Italy (4%), and the United Kingdom (4%). Overall, the geographical distribution of first-author affiliations was heavily concentrated in North America and Western Europe.

Commentaries comprised the majority of publications (54.8%), followed by reviews (15.5%), original research studies (14.3%) and guidelines/consensus statements (14.3%). Only one publication was classified as a policy or legal analysis. Reviews were predominantly narrative in nature, while original research most commonly employed cross-sectional surveys or interviews.

Principal-based reasoning was the most frequently identified ethical framework (44.0%) followed by consequentialist and pragmatic/policy-oriented reasoning (33.3% each). Care ethics was also represented in a substantial minority of publications (20.2%). In contrast, deontological (7.1%), rights-based (3.6%), and virtue ethics (3.6%) frameworks appeared infrequently. Because many publications drew on more than one ethical framework, categories were not mutually exclusive. Explicit mention of ethics-based reasoning was observed in 29 (34.5%) publications, while the remainder employed ethical reasoning implicitly.

Data extraction identified 32 subthemes, which were subsequently organized into 7 overarching themes to enhance conceptual clarity: (1) beneficence and nonmaleficence, (2) respect for persons, (3) equity and accessibility, (4) evidence and trial integrity, (5) governance and stewardship, (6) relational and social impacts, and (7) health system readiness and sustainability (**Table 1**). The frequencies reported in **Table 1** represent the number of included publications in which each theme was identified.

**Table 1:** Organization of literature by ethical themes and subthemes.

Theme	Subthemes	Number of publications	Literature
Beneficence and nonmaleficence	Uncertain clinical benefit	61	(1, 3, 5–8, 10–14, 16, 19–26, 28, 31–34, 36–40, 45–55, 57–59, 61, 62, 65–69, 71, 73–77, 80, 82–84)
	Risk of harm	61	(1, 3–14, 16, 18–20, 22, 23, 25, 26, 28, 30–33, 35–41, 43, 47, 48, 50–59, 61, 64, 66, 67, 69–71, 73–77, 80, 83, 84)
	False hope	12	(20, 21, 23, 27, 34, 43, 46, 56, 60, 77, 79, 82)
	Meaningful benefit	20	(2, 8, 9, 14, 17, 27, 38, 40, 50, 53–55, 59, 61, 63, 65, 67, 69, 75, 77)
	Quality of life	10	(1, 12, 14, 17, 28, 40, 52, 62, 69, 71)
	Medical need	23	(3, 5, 7, 8, 10, 16, 28, 32, 39, 41, 45, 54, 59, 62, 65, 67, 70, 72, 78–80, 83, 84)
	Therapeutic optimism	20	(7, 9, 13, 16, 20, 22, 31, 35, 38, 40, 46, 49, 52–54, 58, 60, 65, 67, 74)
Respect for persons	Informed consent	32	(2, 6, 9, 10, 13–16, 18, 19, 21–23, 25, 27, 30, 35–37, 41, 46, 49, 50, 53, 56, 58, 60, 62, 63, 70, 76, 81)
	Capacity to consent	9	(6, 8, 13, 37, 40, 55, 57, 75, 76)
	Shared decision-making	28	(9, 13–16, 18, 19, 22, 35–37, 40, 45, 46, 52–58, 60, 62, 63, 65, 75, 76, 80)
	Autonomy	28	(2, 6, 8, 9, 12–17, 22, 27, 36, 37, 45, 49, 52, 53, 58, 60, 63, 65, 67, 75–77, 80, 84)
	Clinician obligations	22	(9, 12, 15, 16, 18, 19, 22, 27, 30, 35, 36, 41, 46, 58, 62, 63, 65, 69, 71, 76, 78, 84)
	Health literacy	9	(14, 32, 40, 41, 49, 56, 60, 64, 81)
Equity and accessibility	Trial representativeness	23	(4–6, 11, 16, 19, 22, 25, 28, 29, 31, 39, 41, 48, 50, 51, 55, 57, 61, 68, 69, 80, 83)
	Eligibility restrictions	27	(4–10, 15, 18, 19, 28, 30, 35, 41, 42, 53, 55, 57, 62, 64, 72–74, 78, 80, 81, 83)
	Racial/ethnic disparities	20	(5, 10, 14, 16, 18, 19, 29, 31, 32, 35, 41, 48, 50, 51, 54, 57, 61, 69, 73, 83)
	Cultural acceptability	3	(14, 31, 41)
	Cost and affordability	37	(1, 5, 7–12, 14, 16, 27, 28, 31, 34, 38, 39, 44, 47, 49, 52, 54–56, 59, 62, 64, 65, 69, 71, 73, 74, 77–79, 81, 82, 84)
	Insurance	13	(8, 27, 31, 34, 45, 64, 65, 72, 73, 77, 78, 82, 84)
	Geographic accessibility	12	(2, 9, 10, 14, 31, 35, 41, 55, 57, 71, 74, 81)
	Access to follow-up	20	(6, 9, 10, 13, 14, 26, 31, 38, 41, 42, 56, 57, 62, 64, 67, 73, 74, 77, 78, 81)
Evidence and trial integrity	Amyloid bias	9	(3, 8, 20, 21, 23, 33, 39, 51, 79)
	Surrogate endpoints	12	(7, 16, 23, 33, 36, 43, 54, 61, 66, 79, 80, 82)
	Scientific integrity	16	(4, 20–24, 27, 33, 39, 43, 61, 66, 68, 79, 80, 84)
Governance and stewardship	FDA approval concerns	12	(7, 12, 15, 16, 27, 31, 32, 36, 58, 66, 79, 82)
	Industry influence	9	(22, 23, 27, 33, 34, 43, 68, 79, 82)
	Physician/clinic conflicts of interest	3	(16, 43, 82)
Relational and social impacts	Caregiver burden	10	(2, 6, 12, 14, 38, 40, 50, 69, 71, 77)
	Caregiver well-being	8	(1, 2, 9, 14, 38, 44, 50, 71)
Health system readiness and sustainability	Health system is unprepared	20	(5, 8–10, 14, 15, 18, 31, 32, 35, 41, 42, 52, 62, 67, 71, 74, 76, 78, 81)
	Health system burden	21	(1, 5, 10, 11, 15, 18, 26, 28, 31, 41, 55, 59, 61, 64, 65, 69, 73, 77, 78, 80, 81)
	Misuse of resources	15	(7, 11, 12, 16, 23, 27, 34, 47, 49, 68, 71, 75, 78, 79, 82)

### Beneficence and nonmaleficence

Ethical discussions of beneficence and nonmaleficence were largely characterized by tension between the therapeutic promise of a disease-modifying therapy and uncertainty regarding its clinical value. Although authors acknowledged that mAbs may address a substantial unmet need in AD, uncertain clinical benefit and the potential for serious harm were the most frequently discussed subthemes identified across the literature (*n* = 61 each). Many of these authors questioned whether the modest clinical effects observed in trials justified the risks associated with treatment, including ARIA and other adverse effects. Beyond direct clinical risks and benefits, authors also raised concerns regarding how expected treatment outcomes are communicated; while therapeutic optimism was viewed by some as a potential benefit (*n* = 20), others (*n* = 12) cautioned that overstating likely outcomes could foster false hope and contribute to harm (60, 93). Consequently, the principle of nonmaleficence was given significant weight when balancing these competing factors.

### Respect for persons

Respect for persons was most commonly discussed through the lens of patient autonomy and informed decision-making. Many authors (*n* = 22) argued that clinicians have an ethical obligation to inform patients about mAbs as a potential treatment option regardless of their personal views regarding the therapy’s value. At the same time, clinicians were viewed as responsible for ensuring that patients and their families are adequately informed about the associated risks and uncertainties of treatment through a process of shared decision-making. Some articles (*n* = 2) also presented supported decision-making as a complementary framework, emphasizing that individuals with cognitive impairment should retain ultimate decision-making authority whenever possible (30, 52). Nevertheless, cognitive impairment was recognized as a barrier to informed decision-making alongside the complexity of communicating uncertain benefits and serious risks and varying levels of health literacy among patients (31, 55, 56, 64, 71, 89). Overall, authors framed respect for persons as requiring both patient choice and meaningful support to ensure that treatment decisions are informed and understood.

### Equity and accessibility

Justice and equity concerns across the literature focused on the extent to which patients could realistically access mAbs and the infrastructure required to safely deliver them, including MRI monitoring. Limitations to access were most commonly identified as geographic, structural and financial barriers, with the latter appearing most frequently (*n* = 37). Authors also expressed concerns that restrictive eligibility criteria (*n* = 27) and underrepresentation of diverse populations in clinical trials (*n* = 23) could exacerbate existing health disparities. Disparities encountered by racial and ethnic minority populations and older adults were most prevalent, while three articles also examined the implications of excluding individuals with Down syndrome from trials and treatment (23, 40, 72). Questions regarding the cultural acceptability of mAbs in different healthcare settings were also raised, suggesting that implementation may be challenging in some global contexts (31, 46, 56). Access to mAbs was primarily framed as a matter of distributive justice, emphasizing that equitable use requires more than treatment availability alone.

### Evidence and trial integrity

In research contexts, the strength of the evidence supporting mAbs was frequently debated. Several authors (*n* = 12) argued that using amyloid removal was an insufficient surrogate endpoint for approval, with some (*n* = 9) questioning the validity of the amyloid cascade hypothesis as its scientific basis. Others raised concerns regarding the methodological rigor and integrity of pivotal trials, citing issues such as selection and attrition biases, premature trial termination, and inadequate replication (54, 58, 98, 99). Authors also questioned the reporting and interpretation of trial findings, including incomplete outcome reporting, statistical overinterpretation, and claims of meaningful benefit that are not clearly supported by trial data (38, 39, 48). These concerns were framed as ethical issues because they called into question principles emphasized in the Declaration of Helsinki, particularly the requirements for scientifically valid research and transparency (100).

### Governance and stewardship

Similar to the previous theme, authors questioned whether regulatory, reimbursement, and clinical decision-making processes sufficiently protected public interest. In particular, attention was given to the FDA approval process, with several authors arguing that aducanumab’s controversial approval (*n* = 12) and subsequent concerns regarding advisory board relationships (*n* = 9) created the appearance that industry influence may have been prioritized over evidentiary standards. A smaller number of publications (*n* = 3) extended these concerns to the clinical level, suggesting that physician and institutional financial incentives may also bias treatment recommendations in favor of mAbs (33, 58, 96).

### Relational and social impacts

This was the least frequently discussed theme in the included publications. Despite its limited representation, it reflects principles of care ethics, which emphasize the importance of relationships and responsibilities of care. Discussions emphasized that the ethical implications of mAbs extend beyond individual patients to those involved in their care. Throughout the literature, perspectives were mixed on how these relational considerations apply to mAbs. Some (*n* = 8) argued that mAbs may improve caregiver well-being by preserving patient independence and extending the time they are able to engage meaningfully with loved ones. Others (*n* = 10) cautioned that treatment may introduce additional burdens for caregivers, including the logistical demands of frequent appointments. These views were not mutually exclusive (19, 53).

### Health system readiness and sustainability

Many authors (*n* = 20) pointed out that health systems are unprepared to support the widespread introduction of mAbs and that implementation would likely place additional strain on existing infrastructure and services (*n* = 21). Others (*n* = 15) took a utilitarian approach, arguing that the substantial resources required to develop and maintain mAb-related infrastructure may be better allocated to interventions capable of benefiting a larger proportion of society, such as social support programs or home-based care coverage (49, 65). These concerns were often framed as issues of distributive justice and responsible resource stewardship, particularly in discussions of the feasibility of implementing mAbs in resource-constrained settings, such as parts of South America (22) or Asia (31, 46, 56, 88).

Collectively, the identified themes and their publication-level frequencies suggest that ethical discussions surrounding mAbs extend well beyond questions of clinical benefit and harm. While patient-level concerns—such as safety and informed consent—dominated the included literature, many publications also examined broader issues related to equity and health system preparedness, reflecting an increasingly systems-oriented approach to the ethical evaluation of AD therapies.

## Discussion

This scoping review highlights broad ethical considerations regarding the implementation, prescription, and delivery of anti-amyloid mAbs for AD. The majority of included literature was published within the last five years, demonstrating that the ethical discourse surrounding mAbs has expanded rapidly following the regulatory approval of aducanumab and in parallel with the growing clinical adoption of its successors, shifting from concerns about regulatory and evidentiary uncertainty to challenges of real-world implementation (3, 42, 70, 81). While discussions of concerns frequently aligned with the four bioethical principles (autonomy, beneficence, nonmaleficence, and justice), the literature further examined broader questions of trial integrity, governance, and health system preparedness, suggesting that a comprehensive evaluation of mAbs must simultaneously consider both patient-centered and system-oriented ethical perspectives.

The large number of publications discussing beneficence and nonmaleficence is unsurprising given the controversial evidence base underpinning currently available mAbs, intersecting closely with concerns regarding evidence and trial integrity (39, 54). In this respect, ethical debates surrounding mAbs differ from those around many established therapies because judgments regarding benefit and harm remain inseparable from ongoing scientific disagreements about the validity of amyloid reduction as a surrogate endpoint and how we define clinical meaningfulness (35, 36, 81). Ethical concerns were by no means driven by disagreement about the importance of treating AD, but rather by uncertainty whether modest clinical benefits justified the risks, burdens, and costs associated with treatment. Importantly, the potential harms and benefits of mAbs extend beyond the individual patient to include caregivers, family members, and health systems, underscoring the need to consider the broader consequences of treatment alongside its effects on patients.

Discussions of respect for persons frequently emphasized the influence of informed and shared decision-making on patient autonomy. Autonomy in the context of mAbs is particularly challenging because it is difficult to engage in or even define what constitutes appropriate informed decision-making or consent when there exist many unknowns regarding both benefits and harms (84). Thus, classifying a patient as “informed” requires not merely the disclosure of information but transparency regarding a lack of information, leading to sometimes subjective interpretation of complex and evolving evidence. This places clinicians in the position of supporting autonomous choices while also exercising professional judgment in communicating and contextualizing evolving evidence. Such communication must also preserve space for therapeutic hope without creating overly optimistic expectations that could undermine informed decision-making. The presence of cognitive impairment in AD further complicates decision-making and explains the emphasis on supported decision-making models identified in the literature (30, 52).

Concerns regarding equity suggest that ethical access involves substantially more than regulatory approval or treatment availability. Included publications consistently recognized that financial barriers, MRI requirements, specialist workforce shortages, and restrictive eligibility criteria may disproportionately affect already underserved populations (66, 83). These findings reinforce longstanding concerns that innovations in dementia care risk exacerbating existing health inequities if implementation strategies fail to address structural barriers. Additionally, the frequent discussion of underrepresentation in clinical trials indicates that justice concerns begin at the evidence-generation stage and extend through real-world implementation (21, 44, 63).

An important tension identified across themes was the balance between individual and collective ethical considerations. From the individual perspective, patients and their carers may reasonably value even modest slowing of disease progression (53, 55, 68). However, from a societal perspective, the substantial costs and infrastructure requirements associated with mAbs, in combination with strict eligibility requirements and unrepresentative clinical trials, raise concerns regarding distributive justice and responsible stewardship of healthcare resources. From a utilitarian perspective, many authors argued that limited healthcare resources should be allocated toward interventions capable of generating greater population-level benefit than mAbs (49, 65).

Although ethical concerns were discussed extensively throughout the literature, it is important to note that only one-third of publications explicitly mentioned that ethical frameworks were informing their analyses. This suggests that much of the ethical discourse surrounding mAbs remains implicit, with authors frequently invoking concepts such as autonomy, justice, or beneficence without clearly situating their arguments within established ethical traditions. Greater theoretical transparency may strengthen future scholarship and facilitate more systematic comparison of ethical arguments.

Whether explicit or implicit, the ethical analysis of anti-amyloid mAbs was dominated by principle-based, consequentialist, and policy-oriented frameworks that emphasize risk-benefit assessment, evidence generation, and resource allocation. Care ethics was also represented in a substantial minority of publications, whereas rights-based, deontological, and virtue ethics frameworks were comparatively uncommon. Although this distribution reflects the issues that have received the greatest attention to date, it may leave certain dimensions of treatment underexamined. Anti-amyloid mAb therapy often extends beyond the individual patient to involve caregivers, family members, and multidisciplinary clinical teams through management of treatment-related burdens, shared decision-making, and ongoing monitoring. As a framework that emphasizes relationships, interdependence, and responsibilities of care, care ethics may offer valuable insights into these aspects of treatment that are less visible within traditional risk-benefit analyses (101). Greater engagement with care ethics could therefore complement existing ethical analyses and enrich understanding of how treatment affects those involved in patient care. Similarly, intentional use of rights-based, deontological, and virtue ethics frameworks may broaden ethical analysis by drawing attention to patient entitlements, professional obligations, and the moral qualities that characterize good clinical practice.

### Limitations and future directions

The literature was dominated by commentaries and narrative analyses, with relatively few empirical studies examining stakeholder perspectives or real-world implementation experiences. This highlights the emerging nature of the field and suggests that current ethical discourse remains largely conceptual. Additionally, multiple publications originated from recurring author groups, which may have amplified the visibility of particular themes within the literature. As such, the thematic frequencies reported in **Table 1** represent publication-level counts rather than measures of ethical importance, consensus, or evidentiary strength. Consistent with scoping review methodology, formal quality appraisal was not conducted. Furthermore, the review was restricted to English-language publications and may therefore have excluded relevant ethical perspectives published in other languages. The included literature was also heavily concentrated in North America and Western Europe, raising questions regarding the global applicability of existing ethical analyses. Many concerns identified in this review, particularly those relating to infrastructure, reimbursement, and resource allocation, may manifest differently in low- and middle-income settings where health system constraints are more pronounced. Future work should expand the scope of this literature and incorporate empirical ethics approaches to better understand how patients, caregivers, clinicians, and health systems navigate these challenges in practice.

## Conclusion

Collectively, these findings suggest that ethical evaluation of anti-amyloid mAbs has evolved beyond traditional questions of patient benefit and harm toward broader considerations of evidence generation, equity, and health system sustainability. As potentially disease-modifying therapies become increasingly integrated into clinical practice, ethical analysis will need to balance the interests of individual patients and caregivers with wider societal obligations regarding fairness, transparency, and responsible stewardship of healthcare resources. Empirical studies involving patients, caregivers, and clinicians will be particularly important for understanding how these ethical challenges are negotiated in practice.
