# Supplementary material for: Ethical challenges in the clinical translation of anti-amyloid monoclonal antibodies for Alzheimer’s disease: a scoping review
Source: Front Psychiatry. 2026 Oct 5;17:1953954. doi: 10.3389/fpsyt.2026.1953954 (PMC13678438; doi:10.3389/fpsyt.2026.1953954)
Supplement: Supplementary file 1 [file SupplementaryFile1.docx]

**Appendix A – Full Search Strategy**

The literature search was conducted on 3 March 2026. Searches were performed independently in each database using database-specific syntax. The exact search strategies and the number of records identified in each database are reported below.

PubMed (MEDLINE)

Search strategy:

(((("Ethics"[Mesh]) OR (ethical OR ethic)) OR (ethics OR moral* OR justice OR benefit* OR harm*)) AND (("Alzheimer Disease"[Mesh]) OR (Alzheimer OR Alzheimer's OR dementia))) AND ((((anti-amyloid) OR (amyloid-related)) OR ("recently approved")) OR (((((("lecanemab" [Supplementary Concept]) OR "Antibodies, Monoclonal, Humanized"[Mesh]) OR "donanemab" [Supplementary Concept]) OR "aducanumab" [Supplementary Concept]) OR ("monoclonal antibod*"))) OR aducanumab OR donanemab OR "passive immunotherapy" OR lecanemab OR "biologic therapy" OR "passive immunotherapy")

Filters:

- English
- From 2006 - 2026

Records identified: 744

Scopus

Search strategy:

( ( TITLE-ABS-KEY ( ethic OR ethics OR moral* OR justice OR benefit* OR harm* ) ) AND ( TITLE-ABS-KEY ( alzheimer OR alzheimer's OR dementia ) ) ) AND ( ( TITLE-ABS-KEY ( anti-amyloid OR amyloid-related OR "recently approved" ) ) OR ( ( TITLE-ABS-KEY ( lecanemab OR donanemab OR aducanumab OR "monoclonal antibod*" ) OR TITLE-ABS-KEY ("passive immunotherapy" OR "biologic therapy" ) ) ) )  AND PUBYEAR > 2005 AND PUBYEAR < 2027 AND ( LIMIT-TO ( LANGUAGE , "English" ) )

Records identified: 774

Web of Science

Search strategy:

ethic OR ethics OR moral* OR justice OR benefit* OR harm* (Topic) AND alzheimer OR alzheimer's OR dementia (All Fields) AND anti-amyloid OR amyloid-related OR "recently approved" OR lecanemab OR donanemab OR aducanumab OR "monoclonal antibod*" OR "passive immunotherapy" OR "biologic therapy" (Topic)

Filters:

- Language: English
- Publication years: 2006-March 2026 (implemented using the Web of Science publication-year exclusion filter)

Records identified: 498

Embase

Search strategy:

1, exp ethics/, 420,343

2, (ethics or ethic or moral* or justice or benefit* or harm*).mp. [mp=title, abstract, heading word, drug trade name, original title, device manufacturer, drug manufacturer, device trade name, keyword heading word, floating subheading word, candidate term word], 2,440,257

3, 1 or 2, 2,606,803

4, exp Alzheimer disease/, 319,444

5, (alzheimer's or alzheimer or dementia).mp. [mp=title, abstract, heading word, drug trade name, original title, device manufacturer, drug manufacturer, device trade name, keyword heading word, floating subheading word, candidate term word], 571,447

6, 4 or 5, 571,447

7, ((anti-amyloid or amyloid-related or recently) adj1 approved).mp. [mp=title, abstract, heading word, drug trade name, original title, device manufacturer, drug manufacturer, device trade name, keyword heading word, floating subheading word, candidate term word], 14,090

8, exp monoclonal antibody/, 975,788

9, donanemab/, 892

10, lecanemab/, 1,672

11, aducanumab/, 1,869

12, (monoclonal adj antibod*).mp. [mp=title, abstract, heading word, drug trade name, original title, device manufacturer, drug manufacturer, device trade name, keyword heading word, floating subheading word, candidate term word], 403,673

13, (aducanumab or donanemab).mp. [mp=title, abstract, heading word, drug trade name, original title, device manufacturer, drug manufacturer, device trade name, keyword heading word, floating subheading word, candidate term word], 2,493

14, (passive adj1 immunotherapy).mp. [mp=title, abstract, heading word, drug trade name, original title, device manufacturer, drug manufacturer, device trade name, keyword heading word, floating subheading word, candidate term word], 1,851

15, ((lecanemab or biologic) adj1 therapy).mp. [mp=title, abstract, heading word, drug trade name, original title, device manufacturer, drug manufacturer, device trade name, keyword heading word, floating subheading word, candidate term word], 12,592

16, 7 or 8 or 9 or 10 or 11 or 12 or 13 or 14 or 15, 1,069,450

17, 3 and 6, 43,462

18, 16 and 17, 1,294

19, limit 18 to (english language and yr="2006 -Current"), 1,231

20, limit 19 to "remove medline records", 574

Records identified: 574

Ovid EBM Reviews: Cochrane Central Register of Controlled Trials (CENTRAL) and Cochrane Database of Systematic Reviews (CDSR)
Search strategy:

1, (ethic or ethics or moral* or justice or benefit* or harm*).mp. [mp=ti, ot, ab, fx, sh, hw, kw, tx, ct], 232,109

2, (alzheimer or alzheimer's or dementia).mp. [mp=ti, ot, ab, fx, sh, hw, kw, tx, ct], 29,623

3, 1 and 2, 5,131

4, (anti-amyloid or amyloid-related or "recently approved" or lecanemab or donanemab or aducanumab or "monoclonal antibod*" or "passive immunotherapy" or "biologic therapy").mp. [mp=ti, ot, ab, fx, sh, hw, kw, tx, ct], 16,776

5, 3 and 4,160

Records identified: 160

**Appendix B – Publications included in this review**

|  | **Author (Year)** | **Country of First Author** | **Article Type** | **Study Design** | **Context** | **Type of Ethical Reasoning** | **Principal Findings** |
| --- | --- | --- | --- | --- | --- | --- | --- |
| 1 | Anil et al. (2025) | India | Review | Narrative review | Clinical | Consequentialist | Benefits of lecanemab (QALYs and reduced caregiver burden) are promising but cost-effectiveness remains uncertain. |
| 2 | Araújo et al. (2025) | Portugal | Original research | Survey | Clinical | Care ethics; Consequentialist | Time-saved vs. time-invested discussions need to be part of the informed decision-making process. Most participants in this study found treatment burden acceptable. |
| 3 | Atwood & Perry (2023) | USA | Commentary |  | Clinical | Consequentialist | Limited benefit and significant risk question treatment value. |
| 4 | Banzi et al. (2016) | Italy | Review | Systematic review | Research | Principle-based | Older adults are underrepresented in pivotal trials. |
| 5 | Barbosa et al. (2024) | Brazil | Guideline/Consensus |  | Research | Pragmatic/policy-oriented | Real-world eligibility and global applicability are limited. |
| 6 | Barroeta et al. (2025) | Spain | Commentary |  | Research | Principle-based* | Down syndrome exclusion limits evidence and equity. |
| 7 | Beshir et al. (2022) | UAE | Review | Narrative review | Both | Consequentialist | Aducanumab’s modest benefits are complicated by safety and accessibility concerns. |
| 8 | Beveridge et al. (2024) | USA | Review | Narrative review | Clinical | Consequentialist | Promising therapy with substantial unanswered questions. Broader health system issues need to be addressed in tandem. |
| 9 | Bhalala et al. (2024) | Australia | Commentary |  | Clinical | Care ethics; Consequentialist | Appropriate patient selection and shared decision-making are essential. |
| 10 | Bradshaw & Georges (2024) | Luxembourg | Guideline/Consensus |  | Clinical | Rights-based; Pragmatic/policy-oriented | Equitable access within Europe requires system-wide preparation. |
| 11 | Burke et al. (2023) | USA | Commentary |  | Clinical | Consequentialist | Lecanemab’s classification as a DMT remains questionable amid methodological and cost concerns. |
| 12 | Burroughs & Krain (2023) | USA | Commentary |  | Clinical | Principle-based* | QOL gains remain insufficiently demonstrated. It is difficult to ethically justify the use of aducanumab for the average AD patient. |
| 13 | Burry et al. (2026) | France | Original research | Survey | Clinical | Principle-based; Care ethics | Supported decision-making improves understanding of lecanemab. Care partners’ decisions should not replace patient autonomy, especially when preferences differ. |
| 14 | Chen et al. (2026) | Singapore | Commentary |  | Clinical | Care ethics; Pragmatic/policy-oriented | In Asian regions, a DMT for AD would be particularly valuable. However, regional factors, including cultural norms, caregiving structures, healthcare systems, and resource constraints, shape how mAbs are perceived and implemented. Additionally, greater effort should be made to enroll Asian participants in clinical trials. |
| 15 | Chin et al. (2024) | USA | Commentary |  | Clinical | Care ethics; Deontological | Even if hesitant, geriatricians have a duty to engage with mAbs and memory care more generally as informed patients may wish to pursue treatment. |
| 16 | Chiong et al. (2022) | USA | Guideline/Consensus |  | Clinical | Principle-based; Deontological* | Clinicians should apply the established ethical principles to guide their decisions and discussions about aducanumab. |
| 17 | Cohen et al. (2023) | Canada | Original research | Randomized clinical trial | Research | Care ethics | Lecanemab may preserve health-related quality of life. |
| 18 | Cummings et al. (2021) | USA | Guideline/Consensus |  | Clinical | Principle-based; Pragmatic/policy-oriented | Aducanumab should be limited to carefully selected patients due to uncertain benefits, significant risk of harm, and health system burden. Emphasizes transparent, patient-centered communication to support informed decision-making. |
| 19 | Cummings et al. (2023) | USA | Guideline/Consensus |  | Clinical | Pragmatic/policy-oriented | Lecanemab requires careful selection and monitoring. Emphasizes transparent, patient-centered communication to support informed decision-making. |
| 20 | Daly (2023) | France & Argentina | Commentary |  | Both | Virtue ethics* | Cannot assert that lecanemab is a DMT without recognizing that this is based on a belief about the therapeutic value of amyloid that has not been supported by trial outcomes. |
| 21 | Daly & Kurkinen (2024) | France & Argentina | Commentary |  | Research | Consequentialist | Overstated messaging may undermine informed consent. |
| 22 | Daly (2024) | France & Argentina | Commentary |  | Both | Principle-based* | Ethical use requires transparent communication of uncertainty from clinicians. |
| 23 | Daly et al. (2024) | France & Argentina | Commentary |  | Research | Rights-based* | Language and framing may bias perceptions of benefit. |
| 24 | Daly et al. (2025) | France & Argentina | Commentary |  | Research | Consequentialist; Pragmatic/policy-oriented* | Trial design and reporting raise ethical concerns. |
| 25 | Falgàs et al. (2025) | Spain | Review | Conference proceedings synthesis | Research | Principle-based* | Down syndrome exclusion creates evidence and equity gaps. |
| 26 | Filippi et al. (2026) | Italy | Commentary |  | Clinical | Consequentialist; Pragmatic/policy-oriented | In a European context, sustainable, scalable, and ethical implementation of mAbs within a patient-centered model of dementia care will depend on coordinated policies, equitable resource allocation, and robust real-world registries. |
| 27 | Fleck (2021) | USA | Commentary |  | Both | Principle-based* | Aducanumab represents societal injustice and should be withdrawn from the market. |
| 28 | Frisoni et al. (2025) | Switzerland | Review | Narrative review | Both | Pragmatic/policy-oriented* | Decisions about implementing monoclonal antibody therapies require balancing individual-level benefits with population-level consequences, including costs, feasibility, and equity. The debate reflects fundamental tensions between scientific innovation, clinical practice, and public health priorities. |
| 29 | Gray (2017) | USA | Commentary |  | Research | Principle-based | Minority representation remains inadequate in trials. |
| 30 | Guo & Vaishnavi (2023) | USA | Review | Narrative review | Clinical | Pragmatic/policy-oriented | Accurate diagnosis, patient screening, counseling, and monitoring are increasingly important. |
| 31 | Hazegh Fetratjoo et al. (2023) | Iran | Commentary |  | Clinical | Rights-based; Consequentialist | Major barriers limit feasibility in developing countries. |
| 32 | Heidebrink & Paulson (2024) | USA | Review | Narrative review | Research | Pragmatic/policy-oriented* | Aducanumab highlights failures in trial design, oversight, and regulation. Reform is necessary. |
| 33 | Høilund-Carlsen et al. (2024) | Denmark | Commentary |  | Both | Consequentialist | Benefits are overstated and harms underappreciated. |
| 34 | Hunt et al. (2021) | USA | Commentary |  | Clinical | Principle-based* | Resources should prioritize supportive rather than mAb care. |
| 35 | Jeon et al. (2025) | South Korea | Guideline/Consensus |  | Clinical | Pragmatic/policy-oriented | The KAGP offers forward-looking strategies to support the reasonable and sustainable use of mAbs. mAbs demand careful clinical judgment, clear communication, and a patient-centered approach. Knowing when and how to withhold therapy is just as important as knowing when to initiate it. |
| 36 | Karlawish (2021) | USA | Commentary |  | Clinical | Principle-based; Care ethics | Autonomy must be balanced with trust and education. Financial incentives may be beneficial for maintaining memory centers but may also distort priorities. |
| 37 | Karneboge et al. (2025) | Germany | Commentary |  | Both | Principle-based; Care ethics* | Advances in DMTs are shifting AD care towards more complex decisions; supported decision-making preserves autonomy in early AD. |
| 38 | Katayama et al. (2026) | Japan | Original research | Interviews | Clinical | Care ethics | Care partners describe a complex valuation of mAbs, balancing hope for cognitive improvement and amyloid clearance reassurance against concerns about cost, adverse effects, uncertainty about long-term outcomes, and added burden. Care partners also expressed a willingness to continue medical visits even after clearance, suggesting social support and systems to provide follow-up care are needed. |
| 39 | Kepp et al. (2023) | Denmark | Commentary |  | Both | Consequentialist | Lecanemab's risk-benefit balance is unfavorable, with significant adverse effects (ARIA, possible mortality, and brain changes). Substantial uncertainty surrounds lecanemab due to methodological concerns including unblinding, dropout bias, and subgroup heterogeneity. Trial outcomes do not support the amyloid hypothesis, calling into question the scientific basis for mAbs. |
| 40 | Lawrence et al. (2014) | UK | Original research | Focus groups | Research | Care ethics; Consequentialist* | Participants generally assumed that mAbs would benefit them and downplayed associated risks and potential stigma, unlike carers. Many prioritized diagnostic clarity and maintaining current function. Perception of burden, particularly related to procedures, comorbid conditions, and caregiver responsibilities, played a central role in determining willingness to participate |
| 41 | Lee et al. (2024) | South Korea | Guideline/Consensus |  | Clinical | Pragmatic/policy-oriented | mAbs are a promising therapy for AD, especially in Asia where disease burden is steadily increasing. However, healthcare systems across the continent must prepare for their introduction by establishing new clinical pathways and models of care. This involves increasing health literacy in Asia, training Asian clinicians, building infrastructure, developing culturally acceptable cognitive assessment tools, and increasing Asian representation in trials. |
| 42 | Lee & Stögmann (2025) | Austria | Original research | Epidemiological modeling | Clinical | Principle-based | In Austria, eligible patients greatly exceed system capacity. Health system changes must occur. |
| 43 | Lenzer & Brownlee (2024) | USA | Commentary |  | Both | Pragmatic/policy-oriented | Conflicts of interest may bias regulatory decisions relating to donanemab. Trials have questionable data integrity. |
| 44 | Lin & Neumann (2021) | USA | Commentary |  | Clinical | Consequentialist; Pragmatic/policy-oriented | Prior analyses suggest that mAbs are relatively cost-effective, particularly when evaluated using QALYs; however, these analyses may not fully capture all relevant costs and benefits and therefore may not be comprehensive. |
| 45 | Lundebjerg et al. (2022) | USA | Commentary |  | Both | Principle-based | Aducanumab requires more representative confirmatory trials. Patients should be able to access treatment through informed choice. |
| 46 | Lundebjerg (2024) | USA | Commentary |  | Clinical | Care ethics | Hope should be balanced against realistic expectations. |
| 47 | Maki et al. (2025) | Japan | Commentary |  | Clinical | Consequentialist | Non-pharmacologic approaches may offer greater value than mAbs. |
| 48 | Manly & Deters (2023) | USA | Commentary |  | Research | Consequentialist | Trials of mAbs do not provide adequate evidence of safety or efficacy across racial/ethnic groups. This may increase already existing disparities. |
| 49 | Molinuevo et al. (2016) | Spain | Guideline/Consensus |  | Research | Principle-based* | Preclinical trials raise disclosure and risk-benefit dilemmas. To ethically conduct clinical trials with participants who have AD, we need to improve public understanding of AD using unified vocabulary, resolve the acceptable risk-benefit ratio, and clarify if biomarker status should be disclosed. |
| 50 | Morehouse et al. (2025) | USA | Original research | Survey | Clinical | Care ethics | Caregivers desire coverage for both medication and home-based care but often valued home-based care more than medications. |
| 51 | Padala & Yarns (2022) | USA | Commentary |  | Both | Principle-based; Pragmatic/policy-oriented* | Underrepresentation and high costs may worsen existing disparities. |
| 52 | Parks et al. (2025a) | USA | Original research | Interviews | Clinical | Principle-based; Care ethics | Findings reveal the sensitivity and skill with which clinicians must approach conversations about AD treatment and inform how communication strategies could be adapted for this need. |
| 53 | Parks et al. (2025b) | USA | Original research | Interviews | Clinical | Principle-based; Care ethics* | Clinician communication practices vary and shape shared decision-making. Standardized approaches are needed. |
| 54 | Rabinovici (2021) | USA | Commentary |  | Clinical | Principle-based; Consequentialist | Aducanumab’s approval, while debated, may stimulate broader therapeutic innovation. Shared decision-making is essential. |
| 55 | Ramanan et al. (2023) | USA | Guideline/Consensus |  | Clinical | Pragmatic/policy-oriented | Real-world use of mAbs requires strict patient selection, infrastructure, and on shared decision-making processes that align modest and uncertain benefits with patient preferences, risks, and treatment burdens. Implementation will require expansion of health system capacity to ensure safe and equitable access. |
| 56 | Rentz et al. (2024) | USA | Guideline/Consensus |  | Clinical | Principle-based; Care ethics | Standardized language improves shared decision-making. |
| 57 | Roh et al. (2026) | South Korea | Review | Narrative review | Clinical | Principle-based; Pragmatic/policy-oriented* | Examines ethical challenges of biomarker-driven diagnosis and anti-amyloid therapy, emphasizing uncertainty of benefit, safety risks, consent capacity, and inequities in access and evidence across Korean populations, but particularly highlighting individuals with Down syndrome or serious mental illness. |
| 58 | Rose & Kass (2022) | USA | Policy/legal analysis |  | Clinical | Principle-based; Deontological* | Clinicians may ethically decline treatment, but they are obligated to explain this decision and offer alternatives. |
| 59 | Rossini & Pappalettera (2026) | Italy | Commentary |  | Clinical | Principle-based* | Risk of overtreatment necessitates careful patient selection, balancing individual autonomy with responsible use of healthcare resources. |
| 60 | Rydberg et al. (2025) | USA | Original research | Content analysis | Clinical | Principle-based; Consequentialist | Patients and their families use the Internet to learn about diagnoses and treatment options and use this information to make decisions. Websites detailing mAbs primarily highlight benefits more than risks and omit cost information; conversely, they also usually highlight reductions in cognitive decline as relative rather than absolute. Clinicians should be aware that this potentially influences patient perceptions of treatment. |
| 61 | Salemme et al. (2023) | Italy | Review | Narrative review | Both | Principle-based; Care ethics* | mAbs may extend the length of time patients can maintain autonomy, meaningful relationships, and psychological well-being. However, knowledge gaps and implementation limitations persist; these gaps can no longer be ignored to ensure principles of quality of care. |
| 62 | Salvioni Chiabotti et al. (2025) | Switzerland | Commentary |  | Both | Principle-based; Consequentialist; Pragmatic/policy-oriented* | Rejecting mAbs in Europe would pose ethical and scientific challenges. Excluding APOE4 homozygotes may limit autonomy, though this will ideally change with further research. Approved therapies must have clear, added benefit, and clinical meaningfulness determined through both objective and subjective measures. Safe implementation requires infrastructure and monitoring. |
| 63 | Sarkisian et al. (2024) | USA | Commentary |  | Clinical | Principle-based; Consequentialist* | Some patients may reasonably accept treatment risks. Primary care physicians are well-suited to engage in shared decision-making with these patients. |
| 64 | Sato et al. (2025) | Japan | Original research | Survey | Clinical | Consequentialist; Pragmatic/policy-oriented | Early access to lecanemab in Japan appears feasible, yet infrastructure and financial hurdles remain. |
| 65 | Schindler et al. (2025) | USA | Commentary |  | Clinical | Care ethics | While we strive to develop better therapies, mAbs are a valid option for select patients in fighting a devastating, deadly disease. |
| 66 | Schneider (2022) | USA | Commentary |  | Research | Virtue ethics | The EMERGE and ENGAGE trials of aducanumab were methodologically flawed, including protocol changes, early termination for futility, and reliance on post hoc and selectively reported analyses. The evidence does not demonstrate clinically meaningful benefit, and claims linking amyloid reduction to cognitive improvement are unsupported and potentially misleading given inconsistent and minimal effects. |
| 67 | Schott & Marshall (2025) | UK | Commentary |  | Clinical | Principle-based; Consequentialist | mAbs provide meaningful benefit despite controversy. They could become cost-effective when long-term benefits and broader system impacts are considered. |
| 68 | Scott (2024) | Australia | Commentary |  | Research | Consequentialist | Despite trials of mAbs reporting positive results, the authors conclude that they do not provide high-quality evidence of clinically meaningful impacts at an affordable cost. This is in part due to the design of the trials, which were not representative, balanced, or free from industry influence. |
| 69 | Scrase & Budhwar (2023) | USA | Commentary |  | Clinical | Principle-based; Consequentialist* | mAbs show modest benefits but raise complex questions about efficacy, safety, and equity. To some, they may seem "necessary", but there exists much nuance (e.g., harmful side effects). Cost and access barriers will greatly affect underserved populations. |
| 70 | Shields (2025) | USA | Commentary |  | Research | Principle-based; Deontological | Withholding APOE risk information in trials for mAbs undermines informed consent. |
| 71 | Smith et al. (2025) | Canada | Guideline/Consensus |  | Clinical | Principle-based; Pragmatic/policy-oriented | mAbs have been shown to slow the rate of decline. To implement them in Canada, substantial changes must occur in the organization of dementia care. There are unanswered questions about the use of mAbs within the Canadian healthcare system (e.g., accessibility). |
| 72 | Stern & Selkoe (2022) | USA | Commentary |  | Clinical | Principle-based; Pragmatic/policy-oriented | CMS' coverage restrictions for mAbs to only those in RCTs is unprecedented, holding mAbs to a higher standard than other critical drugs. This will cause unnecessary restrictions on patients. |
| 73 | Tampi (2025) | USA | Commentary |  | Both | Consequentialist; Pragmatic/policy-oriented | Lecanemab's clinical meaningfulness remains debated, and treatment requires extensive infrastructure, monitoring, and patient selection. High costs and variable regulatory decisions highlight the complexity of implementing this therapy. Evidence gaps remain regarding long-term outcomes and applicability among diverse populations. |
| 74 | Tayeb et al. (2023) | Saudi Arabia | Guideline/Consensus |  | Clinical | Pragmatic/policy-oriented | AD is a significant health challenge facing Saudi society. Aducanumab's approval created hope for many; however, there is need for caution. Trials were not representative of Saudi Arabia, and neither is the validation of key assessment tools. High rates of illiteracy may complicate cognitive assessment. Adequate infrastructure does currently not exist. |
| 75 | van Veluw & Young (2024) | USA | Commentary |  | Clinical | Principle-based; Deontological* | Some argue that these modest benefits are sought after to preserve a patient's dignity, yet this implies that those with cognitive impairment experience a loss of dignity, propelling a harmful public narrative. Some may object, saying "dignity" is defined differently in this context; others may say that there are other motivations (e.g., autonomy and independence). |
| 76 | Villain et al. (2022) | France | Review | Narrative review | Clinical | Principle-based* | Regardless of their opinion, clinicians are ethically obligated to inform patients of mAbs. This context necessitates support persons and potentially collective, interdisciplinary decision-making. |
| 77 | Wahl & Gadbois (2024) | USA | Original research | Qualitative study | Clinical | Principle-based; Pragmatic/policy-oriented | Stakeholder debates about mAb coverage reveal fundamental ethical tensions between patient autonomy, evidentiary standards, and responsible stewardship of healthcare resources. |
| 78 | Wahlberg et al. (2024) | Sweden | Commentary |  | Clinical | Pragmatic/policy-oriented* | Traditional reimbursement models are insufficient to address challenges associated with mAbs. Innovative approaches, including performance-based payment models and learning healthcare systems, are proposed to balance early access with uncertainty while enabling real-world data collection. |
| 79 | Walsh et al. (2021) | UK | Commentary |  | Research | Virtue ethics | People with AD and their families need hope, not false hope. Aducanumab set a dangerous precedent. |
| 80 | Walsh et al. (2024) | UK | Commentary |  | Clinical | Consequentialist; Pragmatic/policy-oriented | Based on current evidence, it is unclear whether amyloid immunotherapy can ever significantly reduce population-level dementia morbidity at scale. |
| 81 | Weber et al. (2025) | USA | Commentary |  | Clinical | Pragmatic/policy-oriented | Implementation of lecanemab in health systems is a complex and multidisciplinary process that requires institutional investment for appropriate delivery and monitoring. It may be beneficial for institutions to develop unique delivery models based on their specific infrastructure. |
| 82 | Whitehouse & Saini (2022) | USA & Canada | Commentary |  | Clinical | Consequentialist | Aducanumab should be withdrawn. There should be a shift in focus toward public health approaches to better serve individuals with dementia and society as a whole. |
| 83 | Yadollahikhales & Rojas (2023) | USA | Review | Narrative review | Both | Pragmatic/policy-oriented* | There are several iterations of mAbs for the treatment of AD, each driving major innovation in trial design while introducing ethical challenges. Future clinical trials for mAbs have the opportunity to improve diversity. Amyloid status disclosure to cognitively asymptomatic individuals is a pertinent consideration. |
| 84 | Yeo-Teh & Tang (2023) | Singapore | Review | Narrative review | Research | Principle-based; Deontological* | Aducanumab is associated with modest benefits and significant harm. There should be more stringent and insistent requirements on replication and reproducibility of research prior to approval. |

* Articles that explicitly identified their analysis as ethical

“Commentary” includes commentaries, editorials, letters to the editor, perspectives, viewpoints, essays, and opinion pieces.” Guideline/Consensus” includes clinical practice guidelines, position statements, expert consensus, and appropriate use recommendations.

**Appendix C – Definitions of Ethical Frameworks**

| **Ethical Framework** | **Definition** |
| --- | --- |
| Principle-based | Reasoning based on, or explicit mention of, the four core biomedical principles (autonomy, beneficence, nonmaleficence, and justice). If *only* benefits and harms were discussed, consequentialist was chosen instead. |
| Rights-based | Reasoning grounded in individual, group, or human rights; discusses legal or moral entitlements. |
| Care ethics | Reasoning focused on relationships, vulnerability, and responsibility to care. Heavy emphasis on patient feelings, care partners, or patient-provider relationships. |
| Consequentialist | Evaluation is based on outcomes or consequences; discusses benefits vs. harms, cost-effectiveness, or whole population/health system impacts. |
| Deontological | Reasoning based on duties, rules, or obligations regardless of outcomes. |
| Virtue ethics | Reasoning based on character and virtues of the moral agent; focuses on integrity, honesty, prudence, and trustworthiness. |
| Pragmatic/policy-oriented | Discusses real-world solutions, implementation considerations, and system constraints. |
